# Joint EAES-SAGES framework for developing procedure-specific surgical quality assessment tools

**DOI:** 10.1007/s00464-026-12986-0

**Published:** 2026-07-15

**Authors:** Daniel Campioni-Norman, Aimee K. Gardner, Stella Mavroveli, Dylan J. Grisell, Piers Boshier, Noosha D. Deravi, Ahmad Hider, Courtney Collins, Johanna Brandenburg, Ozanan R. Meireles, Martin Wagner, Brent Matthews, Nicole D. Bouvy, Liane S. Feldman, E. Matthew Ritter, George B. Hanna

**Affiliations:** 1 Department of Surgery and Cancer, Imperial College London, London, USA; 2 Department of Surgery, University of Colorado School of Medicine, Aurora, CO USA; 3 Department of Surgery, McGill University, Montréal, QC Canada; 4 Department of Surgery, Indiana University School of Medicine, Indianapolis, IN USA; 5 Department of General and Gastrointestinal Surgery, Ohio State University Wexner Medical Center, Columbus, OH USA; 6 Center for the Tactile Internet With Human-in-the-Loop (CeTI), TUD Dresden University of Technology, Dresden, Germany; 7 Department of Visceral, Thoracic, and Vascular Surgery, University Hospital Carl Gustav Carus at TUD Dresden University Of Technology, Dresden, Germany; 8 Department of Surgery, Duke University School of Medicine, Durham, NC USA; 9 Department of Surgery, Atrium Health Carolinas Medical Center, Charlotte, NC USA; 10 Leiden University, Leiden, The Netherlands

Surgical training has traditionally relied on surrogate measures of technical competence, such as case numbers, supervising clinician recommendations, and performance in written and oral examinations. Consequently, most surgical training pathways have been time-based rather than competency-based. However, there is a growing shift globally towards competency-based training pathways which emphasise direct observation and assessment of technical and procedural skills.

Competency-based surgical training pathways are now widely adopted, internationally recognised, and increasingly required for accreditation purposes. [1–4] Given the high-stakes nature of competency assessment within surgical training pathways, it is essential that tools used to assess technical skills are developed using rigorous methodologies and demonstrate adequate validity evidence. Despite this need, many existing technical skills assessment tools suffer from poor development practices and lack robust validity data [5, 6].

Although test development theory is well established in fields such as psychology and general education, its application within surgical competency assessment remains inconsistent. Technical skills assessments in surgery face unique challenges, including differing surgical techniques between surgeons and institutions, variation in surgeon competence and heterogeneity of human anatomy. These challenges, combined with the current shortcomings in procedural competency assessment tool (CAT) development, underscore the need for robust and standardised surgical quality assurance (SQA) processes. SQA broadly encompasses test definition, procedure standardisation, CAT development, and implementation [7, 8]. While the development of SQA and CAT has been applied within clinical trials, a structured standardised SQA framework is yet to be published to guide the wider surgical community [9], Tsai et al., 2019; [10] in the systematic development, validation, and application of procedure-specific tools for surgical video assessment.

Currently, there is a fragmented approach to CAT development across surgical societies and institutions. The European Association of Endoscopic Surgeons (EAES) and the Society of American Gastrointestinal and Endoscopic Surgeons (SAGES) have a shared mission to promote collaboration, education, and quality assurance in surgical care. A key component of this, identified by both societies, is the development of CATs both for direct observation and for video-based assessment (VBA). However, each society employs distinct SQA frameworks with no unified best practice approach. This lack of standardisation can hinder the quality, comparability, and broader applicability of assessment tools.

The aim of this study was to establish a unified EAES-SAGES best practice SQA framework for developing procedure-specific CATs.

## Materials and methods

Peer-nominated experts and key stakeholders in SQA and assessment tool development were invited to participate on behalf of EAES and SAGES, forming an SQA-CAT Taskforce. The SQA-CAT Taskforce comprised executive committee members from both societies, professors of surgery, clinicians with expertise in artificial intelligence application in surgery, specialists in SQA, and assessment methodology, as well as psychologists with experience in test development. The President and President Elect of EAES and the President of SAGES were also in attendance.

A two-day meeting was held in London, in May 2025. The objectives of the meeting were threefold: (1) to collaboratively identify guiding principles for the shared SQA framework, (2) to review and identify differences between the existent EAES and SAGES SQA frameworks, and (3) to propose a standardised SQA framework for development of procedure-specific CATs.

Initially the SQA-CAT Taskforce worked to identify key criteria and guiding principles to be considered when developing the shared SQA framework. The meeting was subsequently structured to include a presentation from each society outlining their SQA framework in detail, evidence for the framework and current implementations. This was followed by an iterative process to identify commonalities and differences and align the two frameworks to propose a standardised best practice SQA framework for CAT development.

## Results

### Guiding principles

The SQA-CAT Taskforce agreed on three guiding principles for the development of the shared SQA framework. These were as follows:

1. Balance between methodological rigour and the practical time constraints of CAT development.
2. Feasibility of framework and CAT implementation across varied surgical settings.
3. Adaptability of framework and CATs for artificial intelligence-based competency assessment methods.

### Comparison of EAES and SAGES SQA frameworks

Both EAES and SAGES have developed and implemented their respective SQA-CAT development frameworks aligned with existing SQA methodology and education theory.

The EAES SQA framework has been developed in line with existing SQA methodology from Imperial College London and it is used to develop CATs for a wide range of procedures, including:

- Laparoscopic colectomy [9], Coleman et al., 2011 [11]; [12]
- Transanal total mesorectal excision (Tsai et al., 2019)
- Laparoscopic TME (Curtis et al., 2020)[13]
- Sentinel lymph node biopsy (in total laparoscopic hysterectomy and bilateral salpingo-oopherectomy [14], Obermair et al., 2021)[15]
- Oesophagectomy. [10]
- Gastrectomy. [16]
- Lymphadenectomy (in oesophagectomy). [17]
- Simulation-based therapeutic , ammoplasty. [18]
- Simulation-based open inguinal hernia (unpublished data).

This methodology has undergone iterative refinement through its repeated application, with contributions from a broad range of stakeholders including clinicians, psychologists, psychometricians, and statisticians. Its methodology has been aligned with international standards for the design and evaluation of educational assessments [7]. The EAES Educational Committee has ratified adaptations of this existing methodology to produce the ‘EAES SQA framework’.

SAGES employs a similar robust and standardised approach to CAT development. This process has been shaped by collaboration between senior clinicians and expert psychologists and is also grounded in internationally recognised principles for educational and psychological test design [7]. With the intended use as a summative assessment to inform competency or certification decisions, SAGES has developed similar assessment tools for laparoscopic fundoplication (Ritter et al., 2019) [19], laparoscopic cholecystectomy (Grisell et al., SAGES 2026), and laparoscopic right colectomy in development.

Each SQA framework shared the same broad stages: (i) definition of test specifications; (ii) construct definition; (iii) tool development; (iv) tool pilot; (v) rater training, and (vi) collection of validity evidence.

Several important differences were found in how these stages are interpreted and executed, which can be seen in Table 1, alongside the final agreed protocol stages. Key differences centred on the methodology for construct definition and tool development. A detailed explanation of differences and how they were resolved in the final SQA framework can be found in Digital Appendices 1.

**Table 1:** Comparison of EAES and SAGES Surgical quality assurance frameworks for competency assessment tool development

Stage	EAES Process	SAGES Process	Differences	Unified Process
Define Test Specifications	• Define test rationale and aim through literature review and stakeholder consultation • Statement on skill gap/clinical need • Define test-takers, test-users and scoring procedures	Define statement of purpose of the test: intended users, uses, interpretations, construct to be measured, examinee population, performance level descriptors of the minimally qualified candidate, test format, administration, security, and scoring procedures	Test specifications defined in more detail within SAGES framework	Define statement of purpose of the test: Construct to be assessed, test use and test population. Test rationale (literature review and stakeholder consultation), statement on skill gap/clinical need, interpretations, performance level descriptors of the minimally qualified candidate, test format, administration, security and scoring
Construct Definition	• Hierarchical task analysis • Cognitive task analysis • Thematic analysis • Delphi consensus	• Job task analysis • Cognitive task analysis • Thematic analysis	• Hierarchical versus job task analysis • Delphi after thematic analysis for consensus	• Hierarchical and job task analysis • Cognitive task analysis • Thematic analysis • Delphi consensus
Tool Development	Data from construct definition input into existing template by steering committee	• Task inventory questionnaire (TIQ) • Data from TIQ analysed to define what is input into tool	TIQ to define content inclusion	TIQ Data from TIQ analysed to define inclusion within tool
Tool Pilot	Review tool and scoring descriptors via expert video observation	Review tool and scoring descriptors via expert video observation	–	Review tool and scoring descriptors through expert video observation and discussion
Rater Training	• Develop tool user manual • Define reference video sets with expert agreed scores. Integrated within procedure-specific online training modules • Define rater audit protocol	• Develop tool user manual • Define reference video sets with expert agreed scores. Integrated within procedure-specific online training modules	–	• Develop tool user manual • Define reference video sets with expert agreed scores. Integrated within procedure-specific online training modules • Define rater audit protocol
Validation	Obtain additional sources of validity evidence as appropriate	Obtain additional sources of validity evidence as appropriate	–	Obtain additional sources of validity evidence as appropriate
Monitoring	–	Post-implementation monitoring	Post-implementation monitoring of CATs	Post-implementation monitoring

## Joint EAES-SAGES SQA framework for developing procedure-specific CATs

**Fig. 1:**
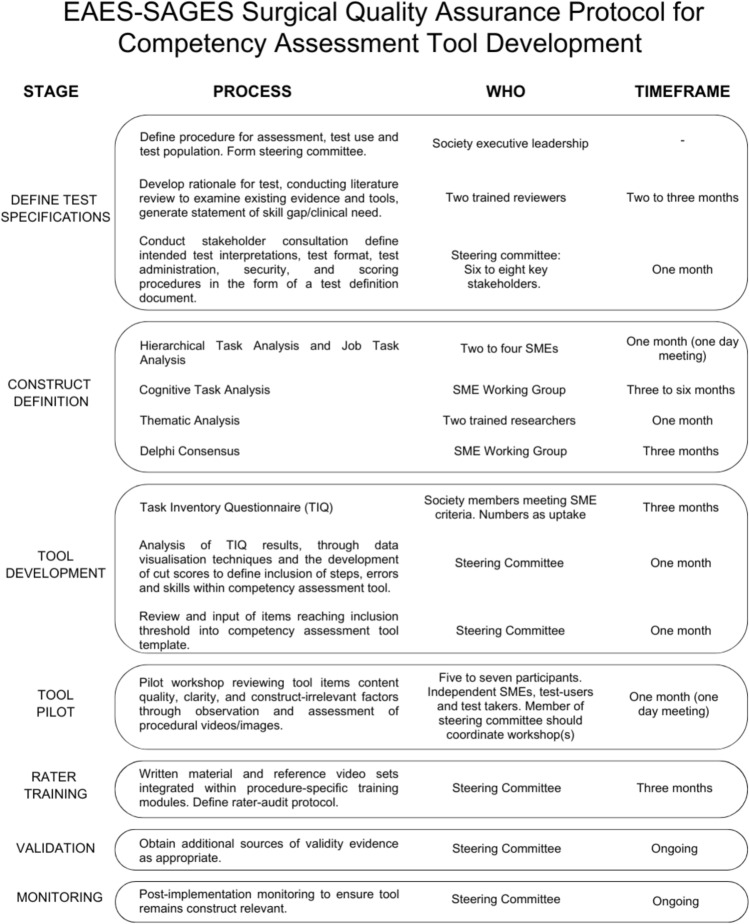
EAES-SAGES Shared Surgical Quality Assurance Protocol for Competency Assessment Tool Development

The shared SQA framework is outlined below, detailing each stage, who within the society should deliver each stage, and the expected timeline. This is concisely summarised in Fig. 1.

### Define test specifications

#### Define procedure, test use, and target population

Initially the procedure to be assessed, the aim of the assessment, and the intended test population should be defined by the society’s or institution’s executive leadership group. This leadership group should appoint a steering committee of six to eight, with a maximum of 10, key stakeholders who will coordinate and implement the SQA framework. Stakeholders should include expert surgeons, educationalists, society representatives, test-takers, as well as AI-experts and surgeons with expertise in AI to ensure the tool could in the future be used to inform automated assessments of competency.

#### Rationale

An evidence-based rationale should be formulated to support the intended use of the test results which will impact the development of the tool. Is the tool designed to provide formative feedback during training? Is it evidence of competence to participate in a surgical randomised trial? Is it a summative assessment to inform competency decisions at the end of training? Once the test use has been defined, a literature review should be conducted to examine existing evidence and ensure no similar tools exist with acceptable validity evidence. This should be conducted by two trained reviewers and take approximately two to three months.

Following the systematic review, a short statement of the target skill gap or clinical problem should be developed, and stakeholder consultation should be undertaken with the steering committee. Stakeholder consultation should seek to define test interpretation, performance level descriptors of the minimally qualified candidate, test format, administration, security, and scoring, summarised in a test definition document. This process should be undertaken within the steering committee and take approximately one month.

Within the SQA framework, procedure-specific expert surgeons will be referred to as subject matter experts or SMEs. SMEs should be selected in line with pre-determined selection criteria such as clinical or academic experience. Ideally, SMEs have registries, databases, or publications demonstrating excellent clinical outcomes. These procedure-specific criteria will be defined by the steering committee at this stage.

### Construct definition

Construct definition is undertaken in an SME-led consensus-driven approach.

#### Hierarchical and job task analysis

Initially a hierarchical task analysis (HTA) is undertaken breaking down surgical interventions into a hierarchical structure of phases, steps, processes, and end-product quality [20]. This is undertaken with two to four SMEs, engaged from more than one society, using a detailed video observational tool such as Behavioural Observation Research Interactive Software (BORIS) (Friard 2016) [21]. Videos should be reviewed by several surgeons and institutions.

Next, a job task analysis (JTA) is undertaken, which is a systematic process undertaken through a workshop with the previous two to four SMEs. Several procedural videos should be reviewed to prompt discussion identifying the specific knowledge, skills, and abilities required to perform each step and process (Wolfe, 1991) [22]. This should take approximately one month.

The HTA and JTA will provide a draft list of procedure phases, steps, process, and end-product quality, alongside a list of knowledge, skills, abilities, and potential risks and errors for each step, forming an interview framework for the cognitive task analysis (CTA). Definitions of each of these terms can be found in Table 2 to support this stage.

**Table 2:** Definitions within the EAES-SAGES surgical quality assurance framework (Hollnagel et al., 2003; Schmidt et al., 2012)

Term	Definition	Surgery specific example
Phase	Significant stages or sections of the overall task, usually grouping of steps	Operative set up
Step	Smaller basic units of a phase. A discrete individual action	Port insertion
Process	The process, or step description, is describing exactly how the step is performed	Perpendicular insertion of trocars with tips visualised throughout
End-product quality	This is the desired outcome or ambition of a step or phase	Trocar insertion with no injury to surrounding structures providing optimal triangulation for procedure
Error	A preventable, unintentional mistake occurring during surgery, which can be consequential or inconsequential	Trocar insertion resulting in visceral injury
Knowledge	Factual, conceptual, or procedural information which can be applied. (What you know)	Knowledge of different suturing techniques
Skill	The performance of specific tasks to accomplish an objective (What you can do)	Suturing
Ability	Capacities and propensities which could be applied to knowledge or skills (What you could do)	Innate dexterity

#### Cognitive task analysis

The CTA is conducted, through semi-structured interviews with an ‘SME Working Group’ (Hollnagel et al., 2003) [20]. SME numbers are guided by procedure novelty, complexity, and test use. These are typically more than 15 in number but can be guided by saturation of the data. The interview is organised according to procedural steps, with prompts for the SMEs to provide missing information, instances of effective performance, instances of ineffective performance, errors experienced or observed, and skills required to perform each step of the procedure. Data are also sought regarding the most critical step for optimal patient outcomes, common reasons to re-intervene, key knowledge, skills and abilities required for performing the procedure successfully, and most challenging aspects of the procedure. Finally, SMEs are asked to indicate the extent to which skills required to complete the procedure might change in the future. The CTA should take approximately three–six months.

#### Thematic analysis

The semi-structured interview data will be examined and organised through a thematic and frequency analysis [23]. This produces a final task analysis capturing all technical variations of the procedure. This will be undertaken by two researchers trained in thematic analysis and should take approximately one month.

#### Delphi consensus

The Delphi will present the final task analysis with all procedure steps and processes, including technical variations, as a list of statements. Members of the ‘SME Working Group’ will individually rate each statement as mandatory, optional, or prohibited. Seventy per cent agreement is recommended based upon existing literature, but this consensus value should be defined by the steering committee as per tool use. After each round, the results and comments from the previous round are presented to the experts.

This process is repeated over multiple rounds until all statements reach the defined consensus value, or a pre-agreed number of rounds have been reached. Any remaining statements after this round should be agreed upon by the steering committee. This generates a robustly defined consensus-derived final list of agreed procedure steps. The Delphi consensus should take approximately three months.

### Tool development

The output of the construct definition stage is a consensus-agreed list of procedure steps, with corresponding SME-derived data from the semi-structured interviews regarding effective/ineffective performance, errors, skills, competencies, criticality, difficulty, and future relevance of each step. The tool development stage seeks to robustly define which steps and associated data should be included within the final assessment tool, undertaken through a Task Inventory Questionnaire (TIQ).

#### Task inventory questionnaire

The TIQ presents the consensus-agreed surgical steps and associated data on errors and skills. SMEs are asked to score each step on importance and difficulty. Errors associated with each step are scored on error importance and error impact on patient outcomes. Finally, SMEs are asked to score the frequency of use and future importance of listed skills.

The TIQ will be undertaken by a wider group of SMEs, recruited internationally through eminent endoscopic surgical societies. Numbers will be guided by society membership and uptake, with previous initiatives recruiting nearly 200 SMEs (Ritter et al., 2019) [19]. This process should take approximately three months.

The steering committee will analyse TIQ results through data visualisation techniques, such as heat-map analysis, and subsequently develop thresholds to robustly define inclusion of steps, errors, and skills within the assessment tool. Inclusion is based upon the following scores from the TIQ:

- **Steps**: Step Importance Score, Step Difficulty Score
- **Error**: Error Importance Score, Error Impact Score
- **Skill**: Skill Frequency Score, Skill Importance Score

#### Populating CAT

The data for inclusion within the tool will be reviewed by the steering committee and input into the CAT template. A CAT follows a standardised format assessing domains of surgical competence against procedure-specific steps. This structured framework was originally developed in an evidence-based approach [9]. For a CAT, each domain is scored one to four, with anchoring single-word descriptors accompanied by detailed behavioural scoring descriptors. The list of consensus-agreed steps, alongside the data generated from the CTA and TIQ, will support scale development and generation of anchoring and detailed scoring descriptors. This should take approximately one month.

The structure of the tool can vary depending on the intended test use, and any amendments should be undertaken in an expert-led approach. Domains could be amended as can the scale. A steering committee may find value in a four-point scale, to provide more detailed formative feedback. For credentialling purposes, the scale could be collapsed to become binary, clearly defining if a desired step, skill or error has been observed. This SQA protocol advises developing a wider scale initially, with the ability to collapse the scale afterwards as required.

### Tool pilot

The CAT should be reviewed for content quality, clarity, an accurate representation of the intended construct, as well as the presence of construct-irrelevant factors. A pilot workshop should be undertaken to facilitate a ‘think-aloud’ discussion to review the tool in a stepwise approach through observation of photographic or video data for each procedure step and domain of the assessment tool. This allows exploration of the cognitive processes behind scoring and addresses any scoring discrepancies, to inform item-level revisions. The pilot workshop may be repeated several times depending on revisions. The workshop should have five to seven participants, including independent SMEs not involved in previous stages, test-users, and test-takers. A member of the steering committee should coordinate the workshop(s). This process should take approximately one month to arrange and complete.

### Rater training

A user manual should be provided describing the test construct, use, population, interpretation, performance level descriptors of the minimally qualified candidate, test format, administration, security, and scoring guidance. Within the user manual, an operation guide should be written detailing the consensus-agreed steps to support and measure clinical compliance for competency assessment. The guide will also serve as a wider educational resource reporting the key data from the consensus process, outlining the key steps, errors, skills, as well as any tips and pitfalls in clinical practice which emerge from the consensus process data.

Alongside this, rater training should be developed, utilising a frame-of-reference approach [24]. This should provide written material and reference video sets with agreed scores to calibrate assessors, integrated within procedure-specific training modules. If there is unanimous agreement within the pilot workshop on a video’s performance level, this can be considered for initial inclusion within the assessor training module. As assessment data are collected, the videos can be refined and amended.

A rater audit protocol should also be implemented, defining acceptable inter-rater agreement thresholds and regular assessment of reliability to avoid rater drift. If this threshold is not met, exploration of the reasoning is required, such as the engagement of a further assessor. Rater refresher sessions may be implemented, guided by the rater audit.

### Validation

The EAES-SAGES SQA framework applies an evidence-based approach to generating validity evidence, aligned with international standards for educational and psychological testing [7]. International standards for educational and psychological testing recommend utilising Messick’s Unified Framework of Validity [25].

**Fig. 2:**
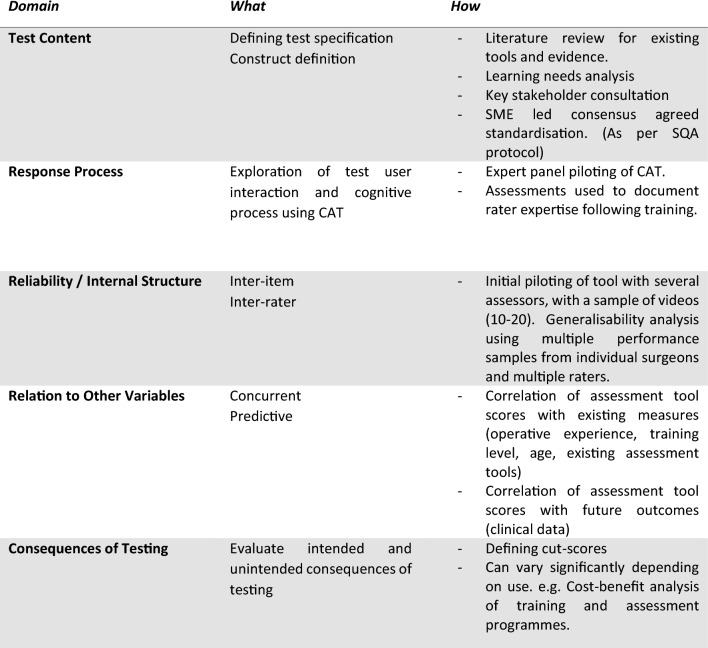
Messick’s Framework of Validity for Surgical Assessment [25]

While this provides a standardised framework for *what* to explore, it is not surgery-specific and does not provide the *how* to explore. This SQA framework provides the how, outlining what to collect, how to collect it and acceptable reporting for each major source of validity evidence (Fig. 2).

## Monitoring

Surgery is a dynamic and rapidly evolving speciality presenting a need for periodical review of content to ensure the CAT still aligns with the desired construct of assessment. This ensures the test content aligns with the procedure construct as it is being practised clinically, accounting for changes in surgical techniques and technologies.

Timeframes should be defined for individual CATs to review and reassess validity evidence to ensure the CAT demonstrates evidence for its intended test use and setting. It is recommended to evaluate how well cut scores are performing on a regular basis. Data should be collected continuously and analysed at least every 3–5 years to inform decisions around the need to modify the assessment tool or rater training.

## Discussion

The alignment of SQA frameworks for CAT Development between two leading international societies, EAES and SAGES, represents a landmark advancement in the standardisation of competency assessment for surgical training. This collaboration reflects a shared mission to promote education, drive quality assurance, and ultimately improve patient care.

As training pathways shift towards competency-based outcome measures, the utility of measuring surgical competence is becoming increasingly important with reported links between surgical performance and patient outcomes, across a range of specialties, using CATs (Balvardi et al., 2022) [26]. This presents significant implications for the use of competency assessment, ranging from summative use in credentialling surgeons for independent practice such as in the UK National Training Programme for Laparoscopic Colorectal Surgery (LAPCO), formative use within surgical training programmes through to defining and maintaining SQA in clinical trials [12, 27]. For competency assessment to inform high-stakes decisions such as credentialling or training progression, there has to be an acceptable level of validity evidence [28]. 

While a large number of surgical competency assessment methods have been described in the literature, the validity evidence supporting their use and scoring interpretations is often limited (Ryan et al., 2022; Vaidya et al., 2019) [5, 6]. One of the key components of validity is ensuring the construct of assessment, what it is we want to assess, is robustly defined and correct. The EAES-SAGES SQA frameworks place a significant focus on construct definition, supporting a detailed breakdown of the technical components of the procedure, alongside underlying knowledge, skills, and abilities to perform each step. This will allow CATs developed in adherence with this framework to demonstrate acceptable validity evidence for construct definition, supporting these tools to inform high-stakes competency decisions.

A key guiding principle underpinning development of the EAES-SAGES SQA framework was ensuring that the framework and resultant CATs demonstrate AI readiness and capability. Within surgical education, AI has seen rapid uptake with emerging applications in personalised training and feedback, automated competency assessment, and adaptive learning trajectories [29].

Importantly, AI readiness should not be viewed solely as the ability to translate existing human assessment tools into automated systems, but rather as a requirement to design assessment frameworks that are inherently compatible with data-driven, scalable, and reproducible evaluation. A central challenge in this transition lies in the translation of subjective, qualitative constructs into objective, quantifiable features suitable for computational modelling.

To address this, the inclusion of surgeons with expertise in the application, development, and evaluation of artificial intelligence, alongside data scientists, within the steering committee enables alignment between surgical domain knowledge and emerging capabilities in computer vision and machine learning. The structured nature of the SQA framework—particularly the use of hierarchical task decomposition, consensus-driven definitions, and standardised scoring systems—provides a strong foundation for generating high-quality annotated datasets required for AI development.

Beyond model development, future work should focus on linking AI-derived metrics to clinical outcomes, ensuring generalisability across institutions, and defining frameworks for human–AI collaboration in assessment workflows.

In this context, the EAES-SAGES SQA framework represents not only a methodology for assessment, but also a foundational infrastructure for scalable, data-driven surgical training and quality assurance.

The alignment of EAES’s and SAGES’s SQA frameworks ensures the adoption of best practices in assessment tool development across both societies. It also facilitates broader implementation, cross-institutional research, and mutual recognition of tools with substantial validity evidence. Importantly, the collaborative framework enables structured feedback from a larger, more diverse community of stakeholders, thereby increasing the credibility, robustness, and acceptability of the tools developed.

The implications for surgical training and assessment are substantial. A shared, evidence-based SQA framework offers a scalable and standardised model for developing valid, reliable, and feasible assessment tools. As surgical education increasingly transitions to competency-based training models, the availability of such rigorously developed tools becomes essential for guiding progression, ensuring credentialing and patient safety outcomes.

While the EAES–SAGES SQA framework provides a structured methodology for developing procedure-specific competency assessment tools, several challenges should be acknowledged. Implementation may require substantial time, expertise, and institutional engagement, which could limit adoption in resource-constrained environments. In addition, the framework relies on access to high-quality procedural video and participation from expert stakeholders across institutions, which may not be uniformly available. Finally, although the framework has been designed with future AI readiness in mind, further methodological work will be required to translate qualitative constructs of surgical performance into robust quantitative metrics suitable for automated assessment.

Despite these challenges, the principal strength of this work lies in the international alignment between two leading surgical societies to establish a transparent, methodologically rigorous, and scalable framework for competency assessment tool development. By integrating expertise from surgical educators, clinical experts, and AI specialists, the EAES–SAGES framework provides a robust foundation for the development of procedure-specific assessment tools that are both psychometrically sound and adaptable to future advances in surgical education and AI-enabled evaluation.

Following a successful and collaborative meeting in London, the immediate next step is the application of the shared EAES-SAGES SQA framework to develop a co-sponsored CAT for laparoscopic inguinal hernia repair. This project will serve as a pilot to test the aligned methodology in a real-world context, allowing refinement based on practical insights and stakeholder feedback. Subsequent phases will focus on expanding the protocol’s application to additional surgical procedures and across other interventional specialties. The long-term aim is to establish the shared EAES-SAGES SQA framework as a global benchmark for surgical competency assessment, ensuring consistency, credibility, and quality across surgical education and practice.

## Conclusion

As surgical training transitions towards competency-based outcome measures, the need for psychometrically sound assessment tools has become increasingly urgent. Current tools often lack sufficient validity evidence to be used confidently in high-stakes settings, with direct implications for both surgeon progression and patient safety.

This white paper presents a co-sponsored, consensus-driven EAES-SAGES SQA framework for CAT Development to support surgical training and assessment. It outlines a unified robust approach to CAT development, grounded in international standards for test development and informed by multidisciplinary expertise. Both EAES and SAGES fully endorse this protocol and are committed to its ongoing application, refinement, and dissemination, ensuring the next-generation surgeons are trained, assessed, and credentialed through transparent, robust, and evidence-based competency outcome measures.

## Supplementary Information

Below is the link to the electronic supplementary material.

Supplementary file1 (DOCX 783 KB)
