# Supplementary material for: Joint EAES-SAGES framework for developing procedure-specific surgical quality assessment tools
Source: Surg Endosc. 2026 Jul 15;40(8):6398–408. doi: 10.1007/s00464-026-12986-0 (PMC13457340; doi:10.1007/s00464-026-12986-0)
Supplement: Supplementary file 1 [file 464_2026_12986_MOESM1_ESM.docx]

**EAES-SAGES Surgical Quality Assurance Framework**

**Digital Appendices**

**Appendices 1**

*Differences Between EAES and SAGES Surgical Quality Assurance Frameworks*

It is apparent both societies methodologies are largely aligned, based upon international standards for educational and psychological testing and engaging key stakeholders such as psychologists and psychometrists (Eignor 2013, Messick 1989). Whilst demonstrating broad alignment, there are subtle but important difference which are discussed below, alongside notable discussion points from the EAES-SAGES meeting.

Construct Definition: Task Analysis Methodology

Defining the assessment construct through task analysis is a foundational step in surgical CAT development, especially important given the procedure-specific heterogeneity inherent in surgical practice. Although both societies prioritise task analysis, they employ different methodologies.

EAES uses a Hierarchical Task Analysis (HTA), a systematic approach that deconstructs a surgical procedure into phases, steps, step descriptions, and end-product quality indicators (Hollnagel, 2003). This process is performed by subject matter experts (SMEs) using a detailed video observational tool, reviewing operative recordings from multiple surgeons across various institutions.

SAGES applies a Job Task Analysis (JTA), which breaks down the surgical procedure into discrete tasks and identifies the knowledge, skills, and abilities required to perform each (Wolfe, 1991). This is conducted by a pre-defined group of SMEs who observe procedural videos and identify task components and contextual variables.

Both SQA protocols subsequently use the results of their respective task analyses to inform the structure and content of Cognitive Task Analysis (CTA) interviews qualitative method used to explore the cognitive processes underlying expert performance (Hollnagel, 2003).

International standards for test development recommend definition of what is being assessed can be derived logically, such as expert opinion, or empirically, such as a detailed video observational tool (Eignor 2013). Each approach offers distinct strengths. HTA provides a highly systematic and standardised method for procedural breakdown, with CTA used to subsequently derive knowledge skills and abilities. JTA, while less systematic approach to defining procedural steps, frontloads valuable insight into the knowledge skills and abilities required for each task, thereby enriching and supporting a more informed approach and framework to the CTA interviews.

The outcome of this was incorporation of a hybrid approach. An initial HTA using detailed video observational tool analysis by SMEs will serve to define procedural steps systematically. This is followed by a focused JTA conducted by small SME groups to explore the knowledge, skills and abilities associated with each step. While these topics will also be addressed during the CTA, early exploration through JTA provides richer data and subsequent framework for the semi-structured interview process.

Construct Definition: Consensus Process

Standardising surgical procedures is a critical component of defining the assessment construct, due to significant variation in procedure-specific technique across surgeons and institutions. Both societies employ consensus processes to establish procedural standards but differ in methodology.

EAES applies the Delphi technique following HTA and CTA. This structured, anonymous process collects expert feedback on procedural steps through iterative rounds of questionnaires. The Delphi method is widely recognised in the literature for minimising bias and enabling broad expert engagement, leading to greater ownership and credibility of the results.

SAGES employs a more interactive, in-person nominal group technique. Experts convene to present, discuss and refine procedural steps until consensus is reached. While this approach fosters fostering a structured environment to facilitate real-time dialogue and idea generation, it is limited by potential bias and logistical constraints, such as smaller participant groups.

Following discussion, both societies agreed to adopt the Delphi method within the shared SQA protocol for procedure consensus. The benefits of reduced bias, increased transparency, and broader expert participation were considered particularly important for ensuring credibility and stakeholder buy-in.

Tool Development

Following robust definition of the construct for assessment, the next stage is using the data collected to populate a structured CAT. Whilst both EAES and SAGES share the goal of translating the construct data into structured, step-based tools assessing surgical competence, they differ in methodology, structure and data integration.

EAES employs a standardised CAT structure in which procedure specific steps are assessed against four domains of competence, established in an evidence-based approach through semi-structured interviews with expert surgeons (Miskovic 2013). This template (Figure 1) is consistently used for CAT development.

**Figure 1**

*EAES Standardised Template for a Competency Assessment Tool*

For a CAT each domain is scored one to four, with anchoring single word descriptors accompanied by detailed behavioural scoring descriptors. The list of consensus agreed steps, alongside the data generated from the cognitive task analysis, will support generation of the anchoring and detailed scoring descriptors. This population of the tool is guided only by the tool development group utilising data from construct definition.

The SAGES protocol uses a similar standardised CAT structure assessing domains of surgical competence against procedure-specific steps. The domains of competence are skill, error and quality. Within each domain of competence for each step there will be a varying number of short statements, which can be scored with varying methodologies depending on the statement. This can include, but is not limited to, a binary yes-no statement, a three-point Likert scale with behavioural scoring descriptors or a checklist format selecting what has been observed within the procedure (Figure 2) (Ritter 2019)

**Figure 2**

*SAGES Example of Competency Assessment Tool*

What distinguishes the SAGES approach is its integration of a Task Inventory Questionnaire (TIQ), which systematically organises data from construct definition into a structured inventory of tasks. A task inventory is a technique which identifies and describes all the tasks, as well as the frequency, importance and difficulty The SAGES TIQ presents the data from the interviews, organising it into major domains to produce an inventory of tasks for the procedure. A wider group of SMEs, identified through predefined selection criteria, are recruited internationally through the eminent endoscopic societies. SMEs are asked to score each step by importance and difficulty. Errors associated with each step are rated on their importance and likelihood to impact patient outcomes. Finally, SMEs are asked to rank the frequency and perceptions of future importance for skills identified for the procedure.

The tool development team will then critically analyse the results of the TIQ, developing cut scores to robustly define inclusion of steps, errors and skills within the CAT. For steps this considers the importance score and difficulty score, for errors this considers the importance score and impact score, and finally for skills this considers the importance score and frequency score. The steps, skills and errors deemed acceptable for inclusion by the cut scores, are considered for inclusion within the tool.

After in-depth discussion, the working group reached consensus on a hybrid approach. The EAES CAT template was selected for the shared protocol, given its consistent structure and four-point scale, which yields richer gradated data for both formative and summative assessment. This scoring scale can be collapsed into a binary format where necessary (e.g., for summative decisions about independent operating). The SAGES Task Inventory Questionnaire (TIQ) methodology was endorsed as a valuable and rigorous process for translating qualitative construct data into quantitatively supported tool components. This will be formally incorporated into the shared SQA protocol to inform tool population decisions.

AI Readiness and Capabilities

Currently, neither the EAES nor SAGES protocols explicitly address the translatability of CATs to AI-based assessment. Given increasing demands on clinical time and the resource-intensive nature of human assessment, both societies recognised the strategic importance of ensuring AI compatibility in future tool development. AI-based assessment offers potential advantages including reducing clinician workload within training and assessment, minimising bias and supporting scalable automated competence assessment (Pedrett 2023).

However, transitioning CATs from human to AI application presents a key challenge: current human-assessment language often centres on observational assessment which relies on qualitative descriptors such as masterful or dangerous, which are difficult to translate into quantitative visual or measurable features required for AI annotation protocols.

The group agreed that **AI considerations must be embedded from the outset** of the development process. **AI-surgeons and AI-experts** should be involved from the **test specification phase**, ensuring alignment with computer vision and machine learning capabilities Qualitative research methods including expert-led workshops and focus groups will be used to translate qualitative descriptors into measurable visual cues and to inform annotation protocols. This approach aims to **bridge the gap between human and AI-based assessment**.

**References:**

Eignor, D. R. (2013). The standards for educational and psychological testing. In *APA handbook of testing and assessment in psychology, Vol. 1: Test theory and testing and assessment in industrial and organizational psychology.* (pp. 245-250). American Psychological Association. <https://doi.org/10.1037/14047-013>

Hollnagel, E. (Ed.). (2003). *Handbook of cognitive task design*. Lawrence Erlbaum Associates.

Messick, S. (1989). Validity. In *Educational measurement, 3rd ed.* (pp. 13-103). American Council on Education.

Miskovic, D., Ni, M., Wyles, S. M., Kennedy, R. H., Francis, N. K., Parvaiz, A., Cunningham, C., Rockall, T. A., Gudgeon, A. M., Coleman, M. G., & Hanna, G. B. (2013). Is competency assessment at the specialist level achievable? A study for the national training programme in laparoscopic colorectal surgery in England. *Ann Surg*, *257*(3), 476-482. <https://doi.org/10.1097/SLA.0b013e318275b72a>

Pedrett, R., Mascagni, P., Beldi, G. *et al.* Technical skill assessment in minimally invasive surgery using artificial intelligence: a systematic review. *Surg Endosc* **37**, 7412–7424 (2023). <https://doi.org/10.1007/s00464-023-10335-z>

Ritter, E. M., Gardner, A. K., Dunkin, B. J., Schultz, L., Pryor, A. D., & Feldman, L. (2020). Video-based assessment for laparoscopic fundoplication: initial development of a robust tool for operative performance assessment. *Surgical Endoscopy*, *34*(7), 3176-3183. <https://doi.org/10.1007/s00464-019-07089-y>

Wolfe, P., Wetzel, M., & Harris, G. (1991). *Job task analysis: Guide to good practice*. Educational Technology Publications.
